# Bearing Fault Diagnosis via Stepwise Sparse Regularization with an Adaptive Sparse Dictionary

**DOI:** 10.3390/s24082445

**Published:** 2024-04-11

**Authors:** Lichao Yu, Chenglong Wang, Fanghong Zhang, Huageng Luo

**Affiliations:** 1 School of Aerospace Engineering, Xiamen University, Xiamen 361102, China; yulichao@stu.xmu.edu.cn (L.Y.); chlowa@stu.xmu.edu.cn (C.W.); 2 National Center for Applied Mathematics, Chongqing Normal University, Chongqing 401331, China

**Keywords:** sparse representation, regularization parameter, stepwise regularization, bearing fault diagnosis

## Abstract

Vibration monitoring is one of the most effective approaches for bearing fault diagnosis. Within this category of techniques, sparsity constraint-based regularization has received considerable attention for its capability to accurately extract repetitive transients from noisy vibration signals. The optimal solution of a sparse regularization problem is determined by the regularization term and the data fitting term in the cost function according to their weights, so a tradeoff between sparsity and data fidelity has to be made inevitably, which restricts conventional regularization methods from maintaining strong sparsity-promoting capability and high fitting accuracy at the same time. To address the limitation, a stepwise sparse regularization (SSR) method with an adaptive sparse dictionary is proposed. In this method, the bearing fault diagnosis is modeled as a multi-parameter optimization problem, including time indexes of the sparse dictionary and sparse coefficients. Firstly, sparsity-enhanced optimization is conducted by amplifying the regularization parameter, making the time indexes and the number of atoms adaptively converge to the moments when impulses occur and the number of impulses, respectively. Then, fidelity-enhanced optimization is carried out by removing the regularization term, thereby obtaining the high-precision reconstruction amplitudes. Simulations and experiments verify that the reconstruction accuracy of the SSR method outperforms other sparse regularization methods under most noise conditions, and thus the proposed method can provide more accurate results for bearing fault diagnosis.

## 1. Introduction

Power transmission is a key segment in driving mechanical equipment, and bearings are one of the most critical parts in transmission systems. With the increasing complexity of modern mechanical equipment structures, the risk of equipment breakdown caused by bearing failure is also increasing. A severe bearing fault may cause huge economic losses or even catastrophic consequences. Therefore, health monitoring is of great significance to ensure the safety and reliability of operational equipment. So far, many techniques for diagnosing bearing faults have been proposed, including vibration monitoring [1,2], acoustic emission inspection [3,4], and thermal analysis [5]. Among them, vibration monitoring techniques, partly due to their maturity in sensing technology and cost-effectiveness, have been widely adopted for bearing fault diagnosis in rotating machinery.

Under constant speed operation, a localized bearing fault will cause a series of periodic transients. Because the interval between adjacent transients is related to the fault location, it is not difficult to locate a fault by identifying the period of repetitive transients. However, actual measurement data usually contains a lot of noise interference, resulting in a quite low signal-to-noise ratio (SNR). How to accurately identify those feature parameters that reflect bearing health conditions from noisy signals is the critical problem to be solved in vibration monitoring based bearing fault diagnosis. Over recent years, many different methods have been proposed by researchers, including empirical mode decomposition (EMD) [6,7], variation mode decomposition (VMD) [8,9], spectral kurtosis (SK) [10,11] and wavelet transform [12]. However, the repetitive transients are sometimes completely submerged in background noise. Accurate extraction of repetitive transient components cannot be ensured through the abovementioned methods, which may lead to false alarms or missing detection, especially in an early stage of fault development when impulse responses are weak [13,14]. Therefore, further improving the accuracy of fault feature extraction from noisy signals is still a research focus of bearing fault diagnosis.

Sparse representation is a powerful signal decomposition technology. By designing an appropriate sparse dictionary, the interested component in signals can be sparsely expressed and accurately reconstructed by a few atoms in the dictionary, while noise cannot. Therefore, sparse representation is characterized by strong de-noising capability. Due to its advantages, more and more researchers are committed to exploring the potential of sparsity-assisted methods in mechanical fault diagnosis [15]. Among them, the sparsity constraint-based regularization method has been extensively researched [16,17,18,19,20,21]. The principle of this method is to transform the repetitive transient extraction into an unconstrained optimization problem by introducing a suitable penalty function as the regularization term and then solving it with optimization algorithms. The L1 norm regularization [22], which obtains the optimal sparse coefficients through minimizing a cost function penalized by the L1 norm, is one of the earliest proposed techniques for sparse regularization. Due to its simplicity in modeling and convenience in calculation, the L1 norm penalty is widely adopted for fault diagnosis. However, the optimal solution of L1 norm regularization tends to underestimate the amplitude of repetitive transients, which may lead to underestimating the severity of bearing faults. To overcome this problem, a generalized minimax-concave (GMC) penalty function [23] was proposed and subsequently applied to fault diagnosis. Wang et al. [24] proposed a regularization method based on the GMC penalty function, and with the method, a higher reconstruction accuracy was obtained as compared to L1 norm regularization in extracting bearing fault signals. He et al. [25] combined the GMC penalty with a tunable Q-factor wavelet transform (TQWT) to enhance the sparsity-promoting capability and achieved good diagnostic results. Huang et al. [26] further extended the GMC regularization to the multi-source sparse representation field and applied it to gearbox compound fault diagnosis. Nevertheless, both GMC regularization and L1 norm regularization face a tricky problem in the selection of the regularization parameter, which is a vital parameter to balance the sparsity and the data fidelity in the sparsity regularized least squares cost function. A larger regularization parameter makes the optimal solution sparser, but the fitting accuracy is lower. On the contrary, a smaller regularization parameter reduces the fitting error but makes the solution less sparse. For conventional sparse regularization methods, generally, the strategy of traversal selection is adopted to determine the optimal regularization parameter, or a k-sparsity method [24] is applied to manually fix the sparsity degree. However, no matter how the regularization parameter is optimized, the optimal solution of the cost function will still be a compromise between pursuing sparsity and reducing fitting error; that is, conventional sparse regularization methods cannot maintain strong sparsity-promoting capability and high fitting accuracy at the same time. Especially in strong interference scenarios, any compromise to sparsity or fitting accuracy may lead to a significant increase in the reconstruction error. As a result, it will be much more difficult to balance both.

In the research field of line spectral estimation problems, Fang et al. [27] proposed an iterative reweighting algorithm that jointly realizes parameter optimization and sparse signal recovery. The method can be used to solve the problem that presumed dictionaries mismatch actual signals and therefore improve reconstruction accuracy. Inspired by this work, in this paper, a stepwise sparse regularization (SSR) method with an adaptive sparse dictionary is proposed, with which strong sparsity-promoting capability and high fitting accuracy are preserved simultaneously, and more accurate signal reconstruction can therefore be realized. Firstly, the time indexes of the sparse dictionary are defined as variables, and a multi-parameter optimization model including time indexes and sparse coefficients is constructed. Secondly, based on the majorization-minimization framework [28,29], sparsity-enhanced optimization is conducted by increasing the weight of the regularization term, making the time indexes adaptively converge to the moments when impulses occur. Meanwhile, by eliminating redundant atoms, the number of atoms in the sparse dictionary can adaptively converge to a number consistent with the exact sparsity degree of actual transient signals. Finally, fidelity-enhanced optimization is carried out by removing the regularization term to obtain high-precision reconstruction amplitudes. A simulation study and two experimental cases validate the effectiveness and accuracy of the proposed method in bearing fault diagnosis.

The remainder of this paper is organized as follows: Section 2 reviews the relevant theories of sparse regularization. In Section 3, the SSR method is derived. Section 4 introduces the parameter setting method and presents the results of the simulation analysis. In Section 5, the proposed method is validated by two practical cases. Finally, the conclusions are summarized in Section 6.

## 2. Theoretical Framework

In this section, the basic principle of sparse regularization and the process of constructing sparse dictionaries are briefly introduced, so as to provide a theoretical basis for the subsequent derivation of the SSR method.

### 2.1. Sparse Regularization

When a localized fault occurs in a bearing, the measured vibration signal usually contains noise interference besides the repetitive transients caused by the damage. According to the previous studies [16], the transient signal can be decomposed into a series of sparse coefficients by a suitable sparse dictionary, so the measured signal $y\in {\mathbb{R}}^{M}$ can be expressed as:

(1) y=Ax+n

where the matrix $A\in {\mathbb{R}}^{M\times N}$ represents the sparse dictionary, $x\in {\mathbb{R}}^{N}$ is a vector consisting of the sparse coefficients, and n denotes the noise interference, which generally complies with Gaussian distribution. Solving the sparse expression of the vibration signal y in the dictionary A can be described as the following optimization problem:

(2) $$\underset{x\in {\mathbb{R}}^{N}}{\min}{\left\|x\right\|}_{0}\;s.t.\;{\|y-Ax\|}_{2}\leq \varepsilon$$

where ${\left\|\;\cdot \;\right\|}_{p}$ denotes the Lp norm and ε is an error tolerance parameter related to noise levels. The optimization above is an NP-hard problem [30,31], which is difficult to be solved directly. By introducing an appropriate penalty function to replace the L0 norm and with the help of regularization, Equation (2) can be further transformed into the following unconstrained optimization problem, which can be solved by using optimization algorithms:

(3) $$\underset{x\in {\mathbb{R}}^{N}}{\min}\;{\left\|y-Ax\right\|}_{2}^{2}+\lambda P\left(x\right)$$

where λ is the regularization parameter, whose value determines the weights of the data fitting term and regularization term in the cost function, thus affecting the value of the global optimal solution. P(x) represents the penalty function that possesses the sparsity-inducing ability. Typical penalty functions include the L1 norm, the GMC function, etc.

### 2.2. Construction of the Sparse Dictionary

Usually, sparse dictionaries are constructed based on prior knowledge. The matching degree between the presumed atoms in sparse dictionaries and actual transient signals greatly affects the reconstruction performance of sparse regularization methods. For transient signals caused by bearing faults, the most commonly used dictionary is Laplace wavelet dictionary, whose form is constructed as:

(4) $$\varphi_{\left(f,\zeta ,\tau \right)}\left(t\right)=\exp\left(\frac{-\zeta }{\sqrt{1-\zeta^{2}}}\cdot 2\pi f(t-\tau )\right)\cdot \sin2\pi f(t-\tau )$$

where f represents the frequency, ζ represents the damping ratio, and τ represents the time index, respectively. Reasonable estimates of f and ζ can be obtained by using the correlation filtering method [32,33], which is to calculate the inner products of the wavelet sequences and vibration signals by traversing all discrete points in the feasible domain of the parameters, and then the one corresponding to the maximum value is the best estimate. The value of τ determines the position of the presumed atom on the time axis. For conventional sparse regularization methods, a common practice is to discretize the time axis into a series of different τ values with the interval of the sampling period. Constructing Laplace wavelet atoms at these positions thus constitutes the atom library of the sparse dictionary A, namely $A=\left[a_{1},a_{2},\ldots a_{N}\right]$, where $a_{n}=\varphi_{\left(f,\zeta ,\tau_{n}\right)}\left(t\right)\in {\mathbb{R}}^{M}$ denotes the n-th column of A, t represents the column vector consisting of all sampling moments. Repetitive transients can be precisely expressed with only a few atoms, so there are actually a large number of redundant atoms in the sparse dictionary.

## 3. Stepwise Sparse Regularization with an Adaptive Sparse Dictionary

Different from conventional sparse regularization methods, which adopt a sparse dictionary with fixed parameters to solve the sparse coefficients, the proposed SSR method optimizes both the time indexes of the dictionary and the sparse coefficients. Therefore, sparsity-enhanced optimization and fidelity-enhanced optimization can be realized step by step. The contradiction between pursuing sparsity and reducing fitting error can be solved, and more accurate reconstructed results can thus be obtained.

### 3.1. Model Establishment

Since the time indexes are defined as variables, the sparse dictionary is no longer a constant matrix, but a function of the time indexes, namely $A\left(\tau \right)=\left[a_{1}\left(\tau_{1}\right),a_{2}\left(\tau_{2}\right),\ldots a_{N}\left(\tau_{N}\right)\right]$, where $\tau =\left[\tau_{1},\tau_{2},\ldots ,\tau_{N}\right]\in {\mathbb{R}}^{N}$, and $a_{n}\left(\tau_{n}\right)=\varphi_{\left(f,\zeta \right)}\left(t,\tau_{n}\right)\in {\mathbb{R}}^{M}$ denotes the n-th column of A(τ). Therefore, Equation (2) changes into:

(5) $$\underset{x\in {\mathbb{R}}^{N},\tau \in {\mathbb{R}}^{N}}{\min}{\left\|x\right\|}_{0}\;s.t.\;{\left\|y-A\left(\tau \right)x\right\|}_{2}\leq \varepsilon$$

In order to solve the problem above and enhance the sparsity-promoting capability, a log-sum penalty function is introduced to replace the L0 norm and establish the cost function to be optimized. Equation (5) is rewritten as:

(6) $$\underset{x\in {\mathbb{R}}^{N},\tau \in {\mathbb{R}}^{N}}{\min}L\left(x\right)=\overset{N}{\underset{n=1}{\sum }}\ln\left({\left|x_{n}\right|}^{2}+\epsilon \right)\;s.t.\;{\left\|y-A\left(\tau \right)x\right\|}_{2}\leq \varepsilon$$

where ϵ is a positive parameter to ensure that the function is well defined. With the help of regularization, Equation (6) can be further transformed into the following unconstrained optimization problem:

(7) $$\underset{x\in {\mathbb{R}}^{N},\tau \in {\mathbb{R}}^{N}}{\min}{\left\|y-A\left(\tau \right)x\right\|}_{2}^{2}+\lambda L\left(x\right)$$

In the proposed method, λ is set as a parameter that varies with the iterative process. The selection principle of λ will be thoroughly illustrated later in this paper. Given the difficulty in directly optimizing Equation (7), the majorization-minimization approach is employed. This approach majorizes the given objective function through the iterative optimization of a simple surrogate function.

### 3.2. Construction and Optimization of the Surrogate Function

${\hat{x}}^{\left(i\right)}=\left[{\hat{x}}_{1}^{\left(i\right)},{\hat{x}}_{2}^{\left(i\right)},\ldots ,{\hat{x}}_{N}^{\left(i\right)}\right]$ and $\lambda^{\left(i\right)}$ are used to denote the sparse coefficients obtained and the regularization parameter selected at iteration i respectively. In the (i+1)th iteration, an appropriate surrogate function $S\left(x|{\hat{x}}^{\left(i\right)}\right)$ which satisfies $S\left(x|{\hat{x}}^{\left(i\right)}\right)-L\left(x\right)\geq 0$ with the equality attained when $x={\hat{x}}^{\left(i\right)}$ is given by:

(8) $$S\left(x|{\hat{x}}^{\left(i\right)}\right)≜\overset{N}{\underset{n=1}{\sum }}\left(\frac{{\left|x_{n}\right|}^{2}+\epsilon }{{\left|{\hat{x}}_{n}^{\left(i\right)}\right|}^{2}+\epsilon }+\ln\left({\left|{\hat{x}}_{n}^{\left(i\right)}\right|}^{2}+\epsilon \right)-1\right)$$

Consequently, the surrogate function for Equation (7) is

(9) $$\underset{x\in {\mathbb{R}}^{N},\tau \in {\mathbb{R}}^{N}}{\min}F\left(x,\tau |{\hat{x}}^{\left(i\right)}\right)≜{\left\|y-A\left(\tau \right)x\right\|}_{2}^{2}+\lambda^{\left(i\right)}S\left(x|{\hat{x}}^{\left(i\right)}\right)$$

According to the majorization-minimization framework, solving Equation (7) now reduces to minimizing the surrogate function iteratively.

Firstly, the constant term in Equation (9) is removed, simplifying it as

(10) $$\underset{x\in {\mathbb{R}}^{N},\tau \in {\mathbb{R}}^{N}}{\min}{\left\|y-A\left(\tau \right)x\right\|}_{2}^{2}+\lambda^{\left(i\right)}x^{T}D^{\left(i\right)}x$$

(11) $$D^{\left(i\right)}=\mathrm{diag}\left\{\frac{1}{{\left|{\hat{x}}_{1}^{\left(i\right)}\right|}^{2}+\epsilon },\ldots ,\frac{1}{{\left|{\hat{x}}_{N}^{\left(i\right)}\right|}^{2}+\epsilon }\right\}$$

where diag{ ⋅ } diagonalizes the vector in parenthesis into a matrix. Given τ fixed, the optimal x of Equation (10) can be obtained by resorting to the Lagrangian multiplier method and given as

(12) $$\tilde{x}\left(\tau \right)={\left(A^{T}\left(\tau \right)A\left(\tau \right)+\lambda^{\left(i\right)}D^{\left(i\right)}\right)}^{-1}A^{T}\left(\tau \right)y$$

Substituting $\tilde{x}\left(\tau \right)$ into Equation (10), the problem above can be further simplified into an optimization of the single variable τ.

(13) $$\underset{\;\tau \in {\mathbb{R}}^{N}}{\min}R\left(\tau \right)≜-y^{T}A\left(\tau \right){\left(A^{T}\left(\tau \right)A\left(\tau \right)+\lambda^{\left(i\right)}D^{\left(i\right)}\right)}^{-1}A^{T}\left(\tau \right)y$$

**Figure 1:**
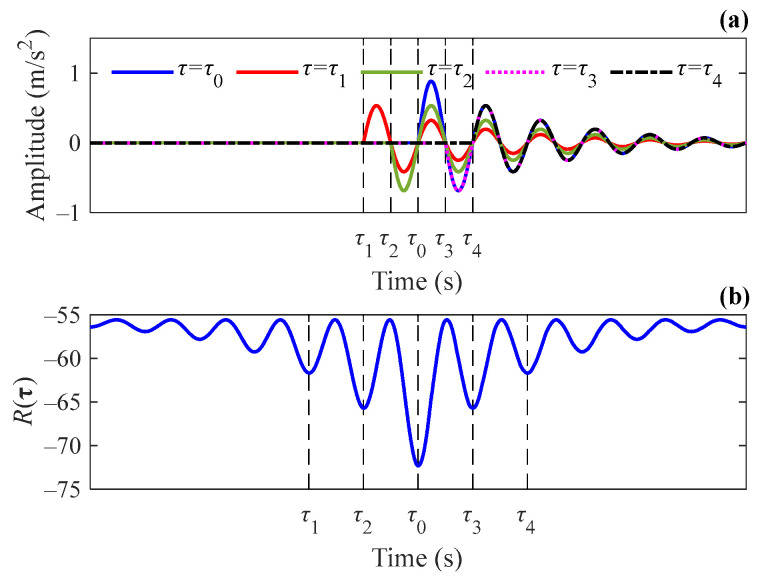
(**a**) The least squares fitting results of an impulse at different moments using Laplace wavelet and (**b**) curves of R(τ) vs. $\tau_{n}$.

Since the object to be optimized is the surrogate function, it is not necessary to find the optimal solution that minimizes Equation (13), but only to search for a suboptimal estimate that decreases the surrogate function. Therefore, such a new estimate for τ can be readily obtained by using the gradient descent method. It should be noted that the waveform of transient signals is characterized by periodic oscillation and thus R(τ) does not decrease monotonically as τ approaches the true value. Assuming that a certain impulse of the bearing fault signal occurs at the moment $\tau_{0}$, when it turns to optimize the corresponding element $\tau_{n}$ of the vector τ, a local minimum of R(τ) can be obtained if and only if $\tau_{n}$ is a multiple of half the oscillation period away from $\tau_{0}$. An example is given in Figure 1a, which shows the least squares fitting results of the impulse using the Laplace wavelet, with the interval between $\tau_{n}$ and $\tau_{0}$ being different multiples of half the oscillation period. Figure 1b shows the trend of R(τ) varying with $\tau_{n}$ near $\tau_{0}$. Generally, it is very difficult to find the global minimum of a function with many extreme points because it is likely to be trapped in a local minimum and thus end the iteration. Fortunately, R(τ) oscillates with a specific period, namely $T_{0}=\frac{1}{2f}$. With this feature, by comparing the values of R(τ) at adjacent positions with the interval being different multiples of $T_{0}$ after each optimization of the gradient descent method, the position that attains the minimum can be set as a new estimate for this round of iteration. Such an optimization process ensures that $\tau_{n}$ gradually converges to the global optimal solution. In other words, as long as a local optimal solution is found, a global optimal solution can be found. Therefore, a new estimate ${\hat{\tau }}^{\left(i+1\right)}$ is obtained by solving Equation (13) with the method above, and the update process for each time index is as follows:

(14) $$\left\{ \begin{array}{l} g_{n}={\hat{\tau }}_{n}^{\left(i\right)}-\alpha \frac{\partial R}{\partial \tau_{n}}\left({\hat{\tau }}^{\left(i\right)}\right)\\ {\hat{\tau }}_{n}^{\left(i+1\right)}=\underset{\tau_{n}\in \left\{g_{n},g_{n}\pm T_{0},\ldots ,g_{n}\pm KT_{0}\right\}}{\min}R\left(\tau \right) \end{array}\right.\;n=1,2,\ldots ,N$$

where $g_{n}$ is the optimized result of the gradient descent method, α is the step size and fixed to 1 in this method, $\frac{\partial R}{\partial \tau_{n}}\left(\tau \right)$ is the first derivative of R(τ) with respect to $\tau_{n}$, K is the maximum multiplier of $T_{0}$ in the second time searching for the optimal estimate, and K=5 is fixed in the proposed method. Here, the corresponding atom $a_{n}\left(\tau_{n}\right)$ in A(τ) need to be updated immediately after $\tau_{n}$ is refined so that the information about the optimized atoms can be used in optimizing $\tau_{n+1}$. The way to obtain the new estimate ${\hat{\tau }}^{\left(i+1\right)}$ ensures that the following inequality holds:

(15) $$R\left({\hat{\tau }}^{\left(i+1\right)}\right)\leq R\left({\hat{\tau }}^{\left(i\right)}\right)$$

Substituting ${\hat{\tau }}^{\left(i+1\right)}$ into Equation (12), the new sparse coefficient estimate is given as:

(16) $${\hat{x}}^{\left(i+1\right)}=\tilde{x}\left({\hat{\tau }}^{\left(i+1\right)}\right)$$

Through iteratively constructing and optimizing the surrogate function, the time indexes gradually converge to the moments when impulses occur, and a more precise estimate of sparse coefficients can therefore be obtained during this process.

### 3.3. Sparsity-Enhanced Optimization and Fidelity-Enhanced Optimization

In order to obtain strong sparsity and high data fidelity simultaneously during the iterative process, different strategies are adopted to choose regularization parameters in different iteration stages, so as to realize sparsity-enhanced optimization and fidelity-enhanced optimization step by step. Referring to the sparse Bayesian learning theory [34,35], the tradeoff between sparsity and data fidelity can be automatically achieved by setting λ to the variance of noise. A reasonable estimate of the noise variance is given by:

(17) $${\hat{\delta }}^{\left(i\right)}=\frac{{\left\|y-A\left({\hat{\tau }}^{\left(i\right)}\right){\hat{x}}^{\left(i\right)}\right\|}_{2}^{2}}{M}$$

In order to emphasize the sparsity-promoting capability in the sparsity-enhanced optimization stage, ${\hat{\delta }}^{\left(i\right)}$ is multiplied by a fixed amplification factor μ, and the product is set as the value of λ in the next iteration to increase the weight of the regularization term.

(18) $$\lambda^{\left(i\right)}=\mu {\hat{\delta }}^{\left(i\right)}=\frac{\mu {\left\|y-A\left({\hat{\tau }}^{\left(i\right)}\right){\hat{x}}^{\left(i\right)}\right\|}_{2}^{2}}{M}$$

For the purpose of maintaining the sparsity obtained in this stage for subsequent optimizations and reducing the computational complexity, the pruning threshold κ is introduced to eliminate the elements smaller than κ in $\hat{x}$ and their corresponding atoms in $A\left(\hat{\tau }\right)$ in each iteration. Thus, the dimension of $A\left(\hat{\tau }\right)$ can be dynamically reduced and gradually converge to the sparsity degree of actual transient signals. Meanwhile, the computing time for updating $A\left(\hat{\tau }\right)$ is shortened.

In fidelity-enhanced optimization, since $\hat{\tau }$ has almost converged to the moments when actual impulses occur, there are no redundant atoms in $A\left(\hat{\tau }\right)$ and each atom corresponds to an actual impulse. Therefore, the sparsity degree of the reconstructed signals can be guaranteed by $A\left(\hat{\tau }\right)$ regardless of the regularization parameter. Without trading off between sparsity and data fidelity, the regularization term can be directly removed by setting λ=0 and the best fitting result can be readily obtained by using the least squares method.

The convergence error at iteration i+1 is defined as $\Delta^{\left(i+1\right)}={{\left\|{\hat{x}}^{\left(i+1\right)}-{\hat{x}}^{\left(i\right)}\right\|}_{2}/\left\|{\hat{x}}^{\left(i\right)}\right\|}_{2}$. It is clear that $\Delta^{\left(i+1\right)}$ will converge to 0 as the iteration results converge. Thresholds $\Delta_{1}=10^{-3}$ and $\Delta_{2}=10^{-6}$ are used to determine the transition from the sparsity-enhanced optimization stage to the fidelity-enhanced optimization stage or the termination of the iteration, respectively. For clarification, the algorithm is summarized as shown in Algorithm 1.
**Algorithm 1.** Pseudo-code of the SSR Algorithm**Input**: vibration signal: y, initial time index: ${\hat{\tau }}^{\left(0\right)}$, M×N sparse dictionary: $A\left({\hat{\tau }}^{\left(0\right)}\right)$, parameters: μ, κ, $\lambda^{\left(0\right)}$

**Initialize**: ${\hat{x}}^{\left(0\right)}=0$, i=0

**Repeat**
    **(1) Construct the surrogate function**
    construct $F\left(x,\tau |{\hat{x}}^{\left(i\right)}\right)$ by Equation (9)     **(2) Optimize the surrogate function**
    **For** n=1,2,…,N, **do**
      update ${\hat{\tau }}_{n}^{\left(i+1\right)}$ by Equations (11), (13) and (14)       update $a_{n}\left({\hat{\tau }}_{n}^{\left(i+1\right)}\right)$ by Equation (4)     **End for**
    **(3) Update the regularization parameter and prune the time index variables**
    update ${\hat{x}}^{\left(i+1\right)}$ by Equations (12) and (16)     $\Delta^{\left(i+1\right)}\leftarrow {{\left\|{\hat{x}}^{\left(i+1\right)}-{\hat{x}}^{\left(i\right)}\right\|}_{2}/\left\|{\hat{x}}^{\left(i\right)}\right\|}_{2}$
    **If** $\Delta^{\left(i+1\right)}>10^{-3}$, update $\lambda^{\left(i+1\right)}$ by Equation (18); **else**, set $\lambda^{\left(i+1\right)}\leftarrow 0$; **end if**
    **For** n=1,2,…,N, **do**
      **If** ${\hat{x}}_{n}^{\left(i+1\right)}<\kappa$, set ${\hat{x}}_{n}^{\left(i+1\right)}\leftarrow \mathrm{null}$; set $a_{n}\left({\hat{\tau }}_{n}^{\left(i+1\right)}\right)\leftarrow \emptyset$; **end if**
    **End for**
   i←i+1

**Until** $\Delta^{\left(i+1\right)}<10^{-6}$

**Return** $A\left({\hat{\tau }}^{\left(i+1\right)}\right){\hat{x}}^{\left(i+1\right)}$

## 4. Simulation Analysis and Parameter Setting

In this section, the effectiveness and accuracy of the proposed SSR method are verified through simulation analysis. Firstly, the influence of related parameters on the performance of the proposed method is studied to provide a basis for the selection of parameters. Secondly, through diagnosing the bearing fault signals generated by simulation and conducting Monte-Carlo analysis, the performances of the SSR, the L1 norm, and GMC methods are compared, and the results validate the advantage of the proposed method in signal reconstruction accuracy.

### 4.1. Generating Simulation Signals

**Figure 2:**
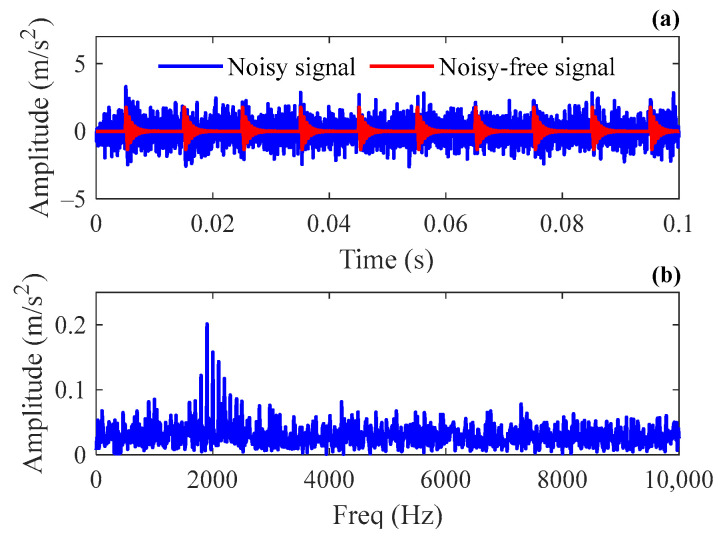
Simulation signal: (**a**) the time domain waveform; (**b**) the frequency spectrum.

The noisy bearing fault signal y(t) is constructed according to the following formula:

(19) $$y(t)=\sum_{\Omega }\left[A\exp\left(\frac{-\zeta }{\sqrt{1-\zeta^{2}}}\cdot 2\pi f(t-\tau_{0}-\Omega T)\right)\cdot \sin2\pi f(t-\tau_{0}-\Omega T)\right]+n(t)$$

where amplitude parameter A=2, damping ratio ζ=0.08, natural frequency f=2000 Hz, time index $\tau_{0}=0.005$ s, and failure period T=0.01 s. The sampling rate is 25,600 Hz, and the signal length is 0.1 s. The noise interference n(t) complies with the Gaussian distribution $N\left(0,\sigma^{2}\right)$ with the standard deviation σ=0.8 and the SNR of the simulation signal can be calculated to be −8.21 dB. Figure 2 shows the graphs of the simulation signal in the time domain and frequency domain. It can be seen that the transient component is almost submerged in background noise. To construct the sparse dictionary, the correlation filtering method is adopted to estimate the parameters ζ and f of Laplace wavelet, and the results $\hat{\zeta }=0.08$ and $\hat{f}=2000$ Hz are consistent with the preset values in the simulation.

### 4.2. Parameter Setting for the SSR Method

The performance of conventional sparse regularization methods is highly dependent on the selection of regularization parameters, while the SSR method is more tolerant to the selection of parameters. The preset parameters in the SSR method include μ, κ, $\lambda^{\left(0\right)}$ and the initial time index ${\hat{\tau }}^{\left(0\right)}$. Among them, the pruning threshold κ is used to eliminate the redundant atoms according to sparse coefficients. Now that transient signals result in large sparse coefficients while noise does not, it is not difficult to find a suitable κ to distinguish the two components. Therefore, κ can be simply set to 5% of the maximal amplitude of measured signals in practical applications. In this method, λ is set as a parameter that is adaptively updated with the iterative process. To improve the stability of the proposed algorithm, the initial value $\lambda^{\left(0\right)}$ is kept unchanged and the time indexes are unpruned during the first few iterations. Obviously, $\lambda^{\left(0\right)}$ only takes effect in the initial stage of iteration and has negligible effect on the sparsity and data fidelity of convergence results. In the proposed method, $\lambda^{\left(0\right)}$ is fixed to 20.

**Figure 3:**
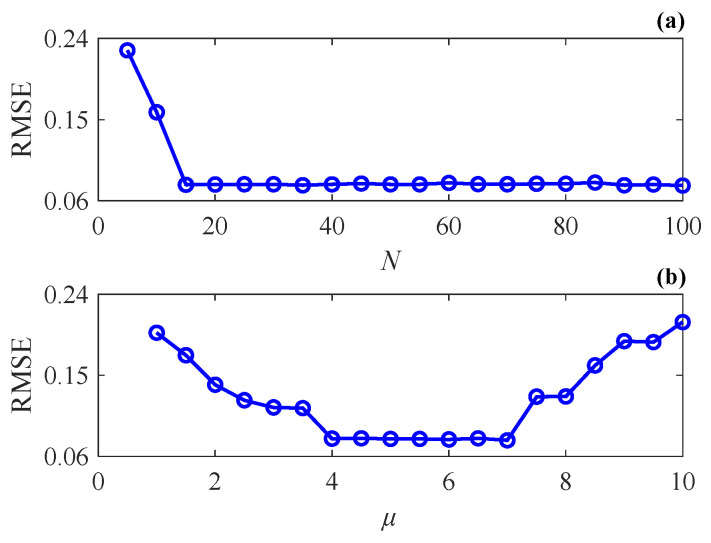
RMSE of the reconstructed signal with different parameters: (**a**) initial atom number N; (**b**) parameter μ.

To determine the value of μ and ${\hat{\tau }}^{\left(0\right)}$, the root-mean-square error (RMSE) is adopted to quantify the effects of the two parameters on signal reconstruction performances. The RMSE is formulated as:

(20) $$\mathrm{RMSE}=\sqrt{\frac{1}{M}\sum_{m=1}^{M}{\left(y_{0}(m)-{\hat{y}}_{0}(m)\right)}^{2}}$$

where $y_{0}$ denotes the noise-free simulated bearing fault signal and ${\hat{y}}_{0}$ denotes the reconstructed fault signal. Since the sparse dictionary in the SSR method changes dynamically, it is not necessary to generate a large number of atoms at the interval of sampling period like that in conventional methods, but only fewer atoms are adequate to reconstruct fault signals precisely. Given μ=5, the number of elements in ${\hat{\tau }}^{\left(0\right)}$, namely the initial atom number N, is changed from 5 to 100 (with augment 5), and all elements are evenly distributed on the time axis. The result is shown in Figure 3a. Because the signal segment contains 10 impulses, it can be seen that once N is larger than the number of impulses, the RMSE decreases to a rather small value rapidly, which means the transient signal is well reconstructed. Besides, the subsequent increase of N has little effect on the RMSE. In practical applications, even if the number of impulses in a signal segment is unknown, the signal reconstruction performance of the proposed method can always be ensured by setting N to a larger value. Accordingly, N=30 is set in the subsequent simulations. For a fixed N, μ is changed from 1 to 10 (with augment 0.5), and the result is shown in Figure 3b. When μ is between 4 and 6, a quite small and stable RMSE can be obtained, while setting μ to other values will lead to a significant increase in RMSE. This is because when μ is too small, the redundant atoms cannot be eliminated completely due to the insufficiency of the sparsity-promoting capability, which leads to an incorrect fitting of transient signals at these positions in the fidelity-enhanced optimization stage. Conversely, when μ is too large, the necessary atoms may also be eliminated due to the overly strong sparsity-promoting capability, which ultimately leads to the absence of some transient components in the reconstructed signal. Based on the above analysis, μ is fixed to 5 in the proposed method.

### 4.3. Simulation Analysis

**Figure 4:**
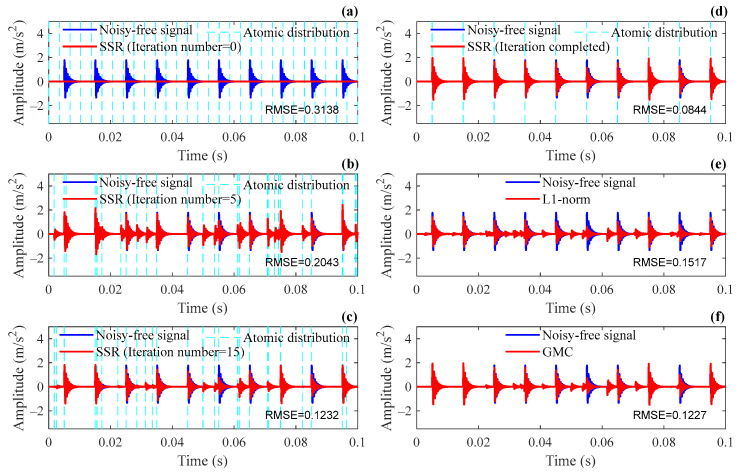
Results of repetitive transients extracted by different methods: (**a**–**d**) the SSR at different iterations; (**e**) the L1 norm; (**f**) the GMC.

The SSR method is used to analyze the simulation signal, and the extracted repetitive transients are presented. The results during and after the iteration are shown in Figure 4. Among them, Figure 4a shows the initial state before the iteration, and Figure 4d is the final result of the SSR, where after 29 iteration steps, the termination criterion has been satisfied. Figure 4b,c show the intermediate steps of the SSR, where the results at iterations 5 and 15 are displayed, respectively, for comparison. In order to demonstrate the dynamic changing process of the sparse dictionary during iterations, the distribution of atoms in the dictionary, namely the coordinates of each element in $\hat{\tau }$, are marked in the figures with dotted lines. It can be seen that the time indexes and sparse coefficients are jointly updated along with the iterative process in the SSR method. The atoms deviating from impulses at first gradually approach the moments when impulses occur. Eventually, the atoms corresponding to impulses are retained, and the redundant atoms are eliminated as the iteration proceeds. The estimate of sparse coefficients can also be more accurate during this process and finally converge to the true value. The simulation results validate that the SSR method is able to extract repetitive transients accurately from noisy signals.

In order to illustrate the improvement of the SSR method in signal reconstruction accuracy, the same segment of simulation signals is analyzed by the L1 norm and GMC methods, and the results are shown in Figure 4e,f. The k-sparsity method is adopted to determine the sparsity degrees in the L1 norm and GMC methods, and the optimal values of parameter k obtained through traversal are k=73 and k=28 respectively. It can be seen from the simulation results that false transient signals appear at some non-impulse moments in both the L1 norm and GMC methods, even when the optimal k is selected, while with the proposed method, more “clean” transient signals can be reconstructed, and it is superior to other methods in terms of amplitude fidelity. The reconstructed result of the SSR method not only achieves the best sparsity degree but also enjoys the lowest amplitude error. According to the value of RMSEs, the improvement of the SSR method over the L1 norm and the GMC method is 44.4% and 31.2%, respectively.

**Figure 5:**
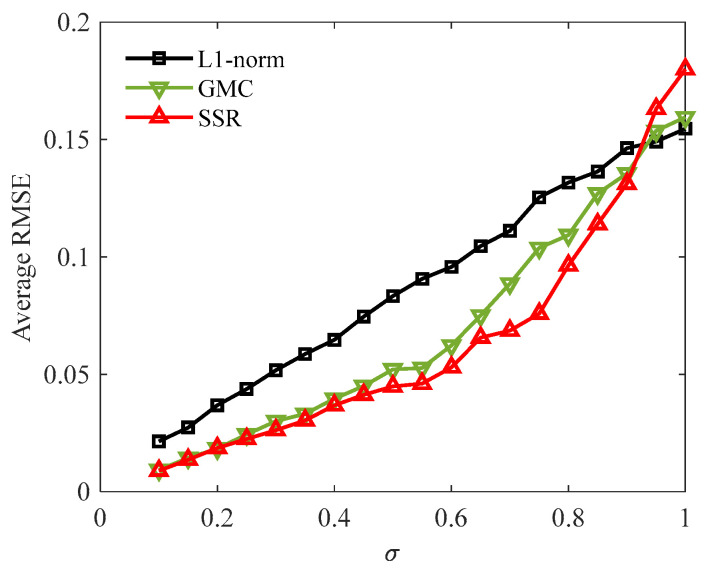
Average RMSEs of different methods varying with noise levels.

To further validate the stability of the SSR method, Monte-Carlo simulations are carried out to explore the signal reconstruction performance under different noise levels. σ is changed from 0.1 to 1 (with augment 0.05), and the average RMSE at each noise level is calculated from twenty repeated experiments. The results of the three methods are shown in Figure 5, where the SSR method achieves the lowest average RMSE under the condition of σ no more than 0.9. Besides, as the noise level increases, the advantage of the SSR method over the other two methods can be observed to increase first and then decrease. This is because it is not difficult to obtain ideal sparsity and data fidelity simultaneously when the noise is small, but with the increase in noise, the tradeoff between sparsity and fitting accuracy becomes increasingly tricky, which makes it more difficult to accurately reconstruct signals. Consequently, the improvement obtained through overcoming this problem becomes gradually remarkable as the noise increases. However, if the noise is large to a certain extent, some atoms may fail to converge to the right positions in the sparsity-enhanced optimization stage; thus, the existing error will be further amplified in the subsequent fidelity-enhanced optimization stage. All in all, the simulation analysis demonstrates that the SSR is a promising method that can render higher signal reconstruction accuracy than other sparse regularization methods in most noise conditions.

Table 1 shows the average computational time of three methods in the cumulative 380 Monte-Carlo trials mentioned above. All calculations were performed on a computer equipped with an Intel Core i9-10850K 3.60 GHz processor. It can be seen that although the computational efficiency of SSR is not as good as that of the L1-norm, it still has a significant advantage over GMC. This is because conventional sparse regularization methods such as GMC construct dictionary atoms at the interval of the sampling period, resulting in large-dimensional matrices. Although the SSR method requires complex iterative operations, it only needs to preset a small number of dictionary atoms to reconstruct all transient components. Therefore, the matrix dimensions are lower, making the computation faster compared to GMC.

## 5. Experimental Verifications

In this section, the effectiveness and accuracy of the SSR method applied to practical fault diagnosis are verified through experimental cases. Vibration data from two bearing fault cases are analyzed by the SSR method to extract the repetitive transients, and the fault characteristic frequencies identified are compared with the nominal ones calculated from bearing geometries. The same signal segments are also analyzed by L1 norm and GMC methods for comparison.

### 5.1. Case 1

**Figure 6:**
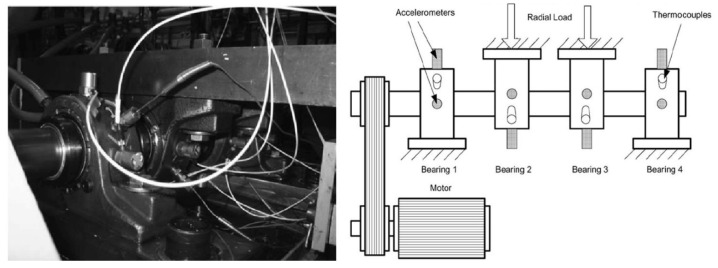
Layout of the bearing test rig.

The data in the first case is the bearing life-cycle dataset from the University of Cincinnati. Figure 6 displays the layout of the bearing test rig, where the shaft is driven by an AC motor rotating at a constant speed of 2000 rpm and coupled by rub belts. Four rolling bearings are installed on the shaft and carry a radial load of 6000 lbs. The sampling frequency is 20.48 kHz, and vibration data were collected every 10 min to generate a segment of 20,480 samples. The dataset consists of a total of 984 files. After 163.8 h of operation, an outer race defect was observed on bearing 1. The bearing designation is ZA-2115, and the fault characteristic coefficient for the outer race is 7.09. Therefore, the fault characteristic frequency can be obtained by multiplying the rotational speed by the fault characteristic coefficient, with its value being $f_{1}=236.4$ Hz. For more details about this experiment, refer to Ref. [36].

**Figure 7:**
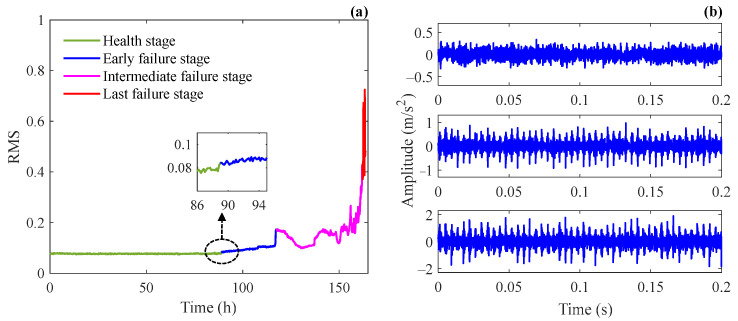
(**a**) RMS of the bearing life-cycle data in Case 1 and (**b**) vibration signals in different stages: early failure stage (upper), intermediate failure stage (middle), and last failure stage (lower).

**Figure 8:**
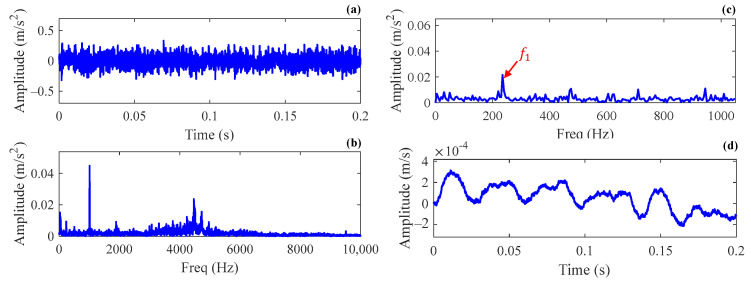
The vibration signal intercepted from the early failure stage in Case 1: (**a**) the time domain waveform; (**b**) the frequency spectrum; (**c**) the envelope spectrum; (**d**) the vibration velocity.

After calculating the root-mean-square (RMS) of all signals in Case 1, the data was divided into healthy stage, early failure stage, intermediate failure stage, and last failure stage according to the variation trend of RMS values as shown in Figure 7a. The upper, middle, and lower signal segments in Figure 7b are vibration signals collected in the early, intermediate, and last failure stages, respectively. It can be seen that signals in the intermediate and last failure stages exhibit strong periodic transients, and the impulses generated in the last failure stage have higher amplitudes, while in the early failure stage, impulses are covered by noise, which makes it difficult to identify the fault feature. Therefore, signals in the early failure stage are selected for bearing fault diagnosis to validate the signal reconstruction capability of the SSR method under heavy background noise. A signal segment with a length of 0.2 s is intercepted from the data collected at the 100th h, and its time domain waveform, frequency spectrum, and envelope spectrum are shown in Figure 8a–c. For reference, the synchronous vibration velocity signal is calculated based on the intercepted acceleration signal and is displayed in Figure 8d, where the RMS value of the vibration velocity is $1.29\times 10^{-4}$ m/s.

**Figure 9:**
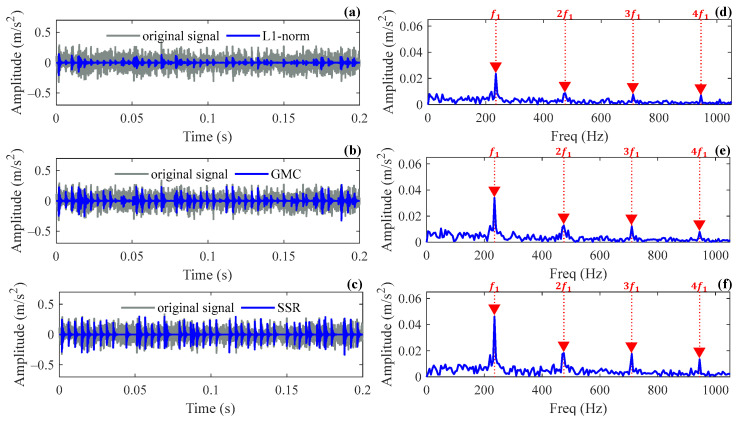
Reconstructed results and their envelope spectrums for different methods in Case 1: (**a**,**d**) L1 norm method; (**b**,**e**) GMC method; (**c**,**f**) SSR method.

The SSR method is used to analyze the intercepted signal. The parameter estimates of the Laplace wavelet obtained through the correlation filtering method are $\hat{f}=4350$ Hz and $\hat{\zeta }=0.06$, respectively. The initial atom number N is set to 60, and the other parameters are consistent with the simulation. The same signal segment is analyzed by the L1 norm and GMC methods for comparison, and the parameter k for both methods is set to 4% of the number of sampling points according to the recommendation. The reconstructed results and envelope spectra of the three methods are shown in Figure 9. It can be seen from the figure that all methods can be used to extract the repetitive transients successfully. Table 2 further compares the sparsity degree of the reconstructed signals and the amplitude (denoted as $X_{f_{1}}$) corresponding to the fault characteristic frequency in the envelope spectrum for the three methods. As can be seen from the table, the SSR method renders the best sparsity degree and the highest reconstruction amplitude over other methods. The experimental results in Case 1 prove that the SSR method is superior to other methods in signal reconstruction accuracy.

### 5.2. Case 2

**Figure 10:**
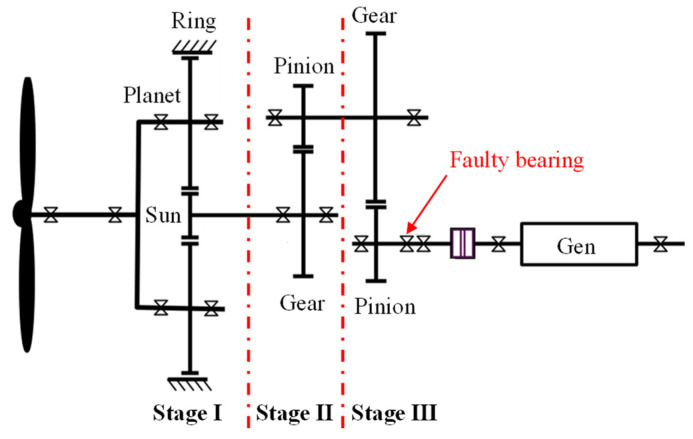
Drivetrain configuration of the wind turbine in Case 2.

**Figure 11:**
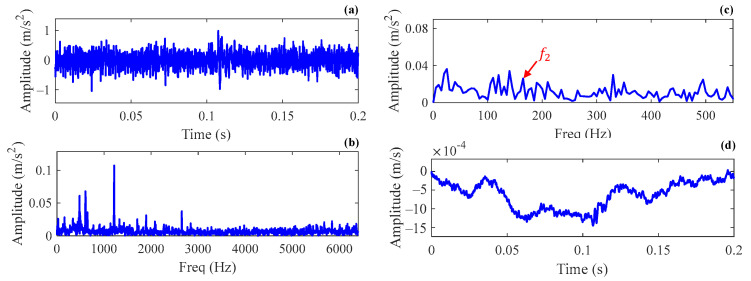
The vibration signal intercepted in Case 2: (**a**) the time domain waveform; (**b**) the frequency spectrum; (**c**) the envelope spectrum; (**d**) the vibration velocity.

This case is the measured vibration data of a wind turbine gearbox in a wind farm. The drivetrain configuration is shown in Figure 10. The rotor speed is augmented by a planetary gear stage and two parallel stages before feeding into the generator. The gearbox has a total gear ratio of 1:78.3. Through inspection, it was found that there was flaking on the inner raceway of the high-speed shaft bearing. The model of the faulty bearing is QJ328N2MA, and its geometry parameters are shown in Table 3. The raw data sampling frequency is 12.8 kHz, and a signal segment with a length of 0.2 s is intercepted for analysis. At the time of data acquisition, the rotating speed of the high-speed shaft is about $f_{r}=23.4$ Hz. Accordingly, the theoretical fault characteristic frequency of the inner race is calculated as $f_{2}=165.2$ Hz. Figure 11a shows the time history of the intercepted vibration signal. The frequency spectrum and envelope spectrum are depicted in Figure 11b and Figure 11c, respectively. The synchronous vibration velocity signal, calculated based on the intercepted acceleration signal, is displayed in Figure 11d. The RMS value of the vibration velocity is $7.36\times 10^{-4}$ m/s.

**Figure 12:**
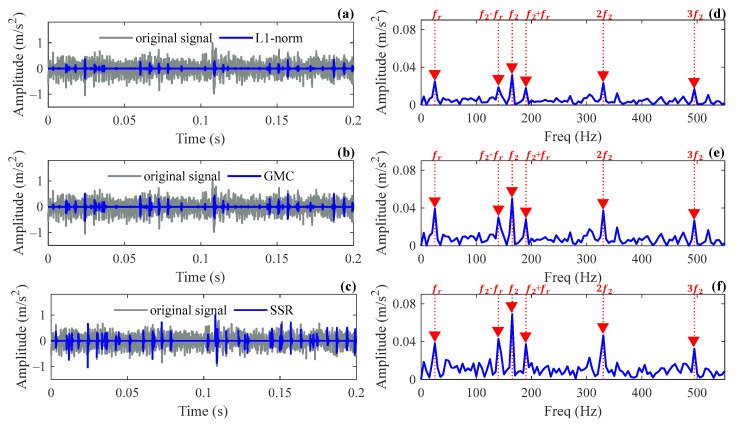
Reconstructed results and their envelope spectrums of different methods in Case 2: (**a**,**d**) L1 norm method; (**b**,**e**) GMC method; (**c**,**f**) SSR method.

The correlation filtering method is used to obtain the Laplace wavelet parameters. The estimates obtained are $\hat{f}=5640$ Hz and $\hat{\zeta }=0.09$, respectively. Then, the intercepted signal is analyzed by the SSR method, where the initial atom number is set as N=60, and the other parameters are consistent with the simulation. The same signal segment is analyzed by the L1 norm and GMC methods for comparison, and the parameter k for both methods is set to 4% of the number of sampling points. The reconstructed results and envelope spectra of the three methods are shown in Figure 12. It can be seen that repetitive transients extracted by the three methods can all indicate the fault characteristic frequency and its harmonics accurately. Additionally, the rotating frequency component as well as the sideband components caused by amplitude modulation can be clearly observed. Table 4 compares the sparsity degree of the reconstructed signals and the amplitude (denoted as $X_{f_{2}}$) corresponding to the fault characteristic frequency in the envelope spectrum, as well as the amplitudes (denoted as $X_{f_{2}-f_{r}}$ and $X_{f_{2}+f_{r}}$) corresponding to the rotating frequency modulation component in the envelope spectrum for the three methods. As can be seen from the table, the SSR method can reconstruct the transient signal with the highest amplitude using significantly fewer atoms compared to other methods. Also, its modulation frequency component is more pronounced than the other methods, indicating that the SSR method not only enjoys optimal sparsity degree but also exhibits an advantage in preserving amplitude fidelity. It has been proven that the signal reconstruction accuracy of the proposed method is better than that of the other two methods.

## 6. Conclusions

In this paper, a stepwise sparse regularization method with an adaptive sparse dictionary is proposed for bearing fault diagnosis. This method addresses the challenge of balancing sparsity enhancement and fitting accuracy improvement in sparse regularization techniques. The strategy is to use a dynamic sparse dictionary to store the information about the location and quantity of transients identified in the iterative process and to reconstruct the transient signal by implementing sparsity-enhanced optimization and fidelity-enhanced optimization step by step.

Compared to the conventional L1 norm and GMC methods, the proposed SSR method offers the following advantages: (1) strong sparsity-promoting capability and high fitting accuracy can be maintained simultaneously, thereby enhancing the signal reconstruction accuracy under most noise conditions; (2) the regularization parameter can be adaptively updated with iterations, and the only parameter that needs to be preset, namely the initial atom number, can be readily determined to ensure the best signal reconstruction performance. The superiority of the SSR method is validated through numerical simulations and two practical applications.

While this paper focuses on the benefits of the SSR as a bearing fault diagnosis method, its superior signal reconstruction capability suggests potential applications in other areas. Future work will investigate extending the SSR method to additional fields.

## Figures and Tables

**Table 1:** The average time cost comparison for the simulated procedures (380 trials).

Method	L1-Norm	GMC	SSR
Average time cost	22.1 s	195.1 s	71.4 s

**Table 2:** The reconstruction result comparison for Case 1.

Method	L1-Norm	GMC	SSR
${\left\|x\right\|}_{0}$	163	163	48
$X_{f_{1}}$	0.0235	0.0339	0.0461

**Table 3:** The geometry of the faulty bearing in Case 2.

Parameters	Value
No. of rolling elements	12
Inside diameter (mm)	140
Outside diameter (mm)	300
Thickness (mm)	62
Pitch diameter (mm)	220
Roller diameter (mm)	47.4
Contact angle (deg)	35
Fault characteristic coefficient for inner race	7.06

**Table 4:** The reconstruction result comparison for Case 2.

Method	L1-Norm	GMC	SSR
${\left\|x\right\|}_{0}$	101	101	49
$X_{f_{2}}$	0.0313	0.0499	0.0691
$X_{f_{2}-f_{r}}$	0.0189	0.0301	0.0429
$X_{f_{2}+f_{r}}$	0.0178	0.0279	0.0373

## Data Availability

Dataset available on request from the authors.
